# Diagnostic model constructed by five EMT-related genes for renal fibrosis and reflecting the condition of immune-related cells

**DOI:** 10.3389/fimmu.2023.1161436

**Published:** 2023-05-17

**Authors:** Yangyang Guo, Ziwei Yuan, Zujian Hu, Yuanyuan Gao, Hangcheng Guo, Hengyue Zhu, Kai Hong, Kenan Cen, Yifeng Mai, Yongheng Bai, Xuejia Yang

**Affiliations:** ^1^ Department of General Surgery, The First Affiliated Hospital of Ningbo University, Ningbo, China; ^2^ Key Laboratory of Diagnosis and Treatment of Severe Hepato-Pancreatic Diseases of Zhejiang Province, The First Affiliated Hospital of Wenzhou Medical University, Wenzhou, China; ^3^ Department of Pathology, The First Affiliated Hospital of Wenzhou Medical University, Wenzhou, China; ^4^ The Affiliated Hospital of Medical School of Ningbo University, Ningbo, China

**Keywords:** renal fibrosis, diagnostic biomarkers, machine learning, immune cell, EMT

## Abstract

**Background:**

Renal fibrosis is a physiological and pathological characteristic of chronic kidney disease (CKD) to end-stage renal disease. Since renal biopsy is the gold standard for evaluating renal fibrosis, there is an urgent need for additional non-invasive diagnostic biomarkers.

**Methods:**

We used R package “limma” to screen out differently expressed genes (DEGs) based on Epithelial-mesenchymal transformation (EMT), and carried out the protein interaction network and GO, KEGG enrichment analysis of DEGs. Secondly, the least absolute shrinkage and selection operator (LASSO), random forest tree (RF), and support vector machine-recursive feature elimination (SVM-RFE) algorithms were used to identify candidate diagnostic genes. ROC curves were plotted to evaluate the clinical diagnostic value of these genes. In addition, mRNA expression levels of candidate diagnostic genes were analyzed in control samples and renal fibrosis samples. CIBERSORT algorithm was used to evaluate immune cells level. Additionally, gene set enrichment analysis (GSEA) and drug sensitivity were conducted.

**Results:**

After obtaining a total of 24 DEGs, we discovered that they were mostly involved in several immunological and inflammatory pathways, including NF-KappaB signaling, AGE-RAGE signaling, and TNF signaling. Five genes (COL4A2, CXCL1, TIMP1, VCAM1, and VEGFA) were subsequently identified as biomarkers for renal fibrosis through machine learning, and their expression levels were confirmed by validation cohort data sets and *in vitro* RT-qPCR experiment. The AUC values of these five genes demonstrated significant clinical diagnostic value in both the training and validation sets. After that, CIBERSORT analysis showed that these biomarkers were strongly associated with immune cell content in renal fibrosis patients. GSEA also identifies the potential roles of these diagnostic genes. Additionally, diagnostic candidate genes were found to be closely related to drug sensitivity. Finally, a nomogram for diagnosing renal fibrosis was developed.

**Conclusion:**

COL4A2, CXCL1, TIMP1, VCAM1, and VEGFA are promising diagnostic biomarkers of tissue and serum for renal fibrosis.

## Introduction

Fibrosis accounts for 45% of all deaths in the industrialized world (1) and can accumulate in multiple organs, such as the heart, lungs, kidneys, and liver. Numerous organ problems may eventually result from extensive tissue remodeling and fibrosis (2). Renal fibrosis is characterized by abnormal accumulation and deposition of extracellular matrix, the occurrence of glomerulosclerosis and renal interstitial fibrosis, which ultimately lead to end-stage renal disease (3). Renal fibrosis may be caused by a variety of damaging causes. The concept of a renal fibrosis niche has been put forth in studies and contends that a range of cells and molecules collaborate to produce a unique pro-fibrosis microenvironment (4). It has been discovered that a range of cells, including macrophages and muscle fibroblasts, contribute to the development of renal fibrosis (3). The imbalance in the proportion of immune cells and the change of state has an important effect on renal fibrosis (5, 6).

Many diagnostic methods for renal fibrosis have emerged. Renal biopsy is the gold standard for the diagnosis of renal fibrosis. However, this approach has several disadvantages, including invasiveness, complexity, and hysteresis (7). Although an innovative non-invasive method to diagnose renal fibrosis has been described (8), its clinical use still requires more verification. VI chain fragments, such as endotrophic protein (PRO-C6), C6M, and C6Mα3, were increasingly expressed in a variety of fibrotic diseases and emerged as key indicators for the detection of fibrosis status (9). Hsa_circ_0036649 expression was found to be reduced in patients with renal fibrosis through the detection and analysis of exosome secretion in the urine for renal fibrosis patients (10). Previous studies revealed that serum lysyl oxidase increased after renal fibrosis, and the enhancement was significant in patients with moderate and severe renal fibrosis (11). However, considering the key role of EMT in renal fibrosis, the diagnostic role of EMT-related genes in renal fibrosis is still unclear.

The EMT has been identified to be a significant driver of organ fibrosis and tumor progression (12). It is known that many small molecule inhibitors have good anti-tumor effects by antagonizing EMT, but their therapeutic effects on fibrotic diseases are not fully understood (13–17). In this study, we used bioinformatics techniques to explore the EMT-related DEGs in healthy human tissue samples and renal fibrosis samples and applied three machine learning algorithms to filter and identify candidate diagnostic markers for renal fibrosis. In-depth analysis was held regarding the potential role of these new diagnostic genes in renal fibrosis, their interaction with infiltrating immune cells, their relationship with anti-tumor drug sensitivity, and their diagnostic efficiency. To satisfy the clinical need for early detection and therapy, the quest for efficient non-invasive diagnostic molecular markers is beneficial for the quick and accurate diagnosis of renal fibrosis.

## Methods

### Data collection and processing

Expression data for renal fibrosis were downloaded from the GEO database. The training set, GSE76882, contained 175 samples of renal fibrosis and 99 control samples. The validation set, GSE22459, had 40 samples of renal fibrosis and 25 control samples. All samples were normalized for subsequent analysis. GSEA (https://www.gseamsigdb.org/gsea/index.jsp) was used to retrieve 201 EMT-related genes, as listed in **Supplementary Table S1**. These genes were downloaded from the gene set “HALLMARK_EPITHELIAL_MESENCHYMAL_TRANSITION”.

### DEGs screening, protein-protein interaction network and enrichment analysis

First, the R package “limma” was used with the screening criterion of p<0.05 to identify the DEGs associated with EMT between the control group and the renal fibrosis group. Next, we plotted PPI network using an online website (STRING: functional protein association networks (string-db.org)). Then, using the R packages “clusterProfiler,” “org.Hs.eg.db,” and “DOSE,” GO and KEGG enrichment analyses for DEGs were carried out.

### Identification and validation of diagnostic markers

Three machine learning algorithms were conducted to identify potential diagnostic genes for renal fibrosis, named least absolute shrinkage and selection operator (LASSO) logistic regression, random forest tree (RF), and support vector machine-recursive feature elimination (SVM-RFE). For LASSO analysis, the R package “glmnet” was utilized. The RF algorithm was used to identify the top 10 significant genes and the SVM-RFE algorithm was used to find the optimal variables. Candidate diagnostic genes were selected when the three machine learning results were intersected, and the expression of these genes was confirmed by RT-qPCR.

### Examining the relationship between immune cells and candidate genes

The CIBERSORT algorithm was applied to assess the contents of 22 immune cells. R software was used to perform the Spearman rank correlation analysis. The R package “ggplot2” was implemented to visualize the associations between candidate genes and various immune cells.

### Gene set enrichment analysis (GSEA)

GSEA was used to investigate potential biological functions of candidate genes. The reference gene set was “c2. Cp. Kegg. V7.0. Symbols. GMT” from The Molecular Signatures Database (MSigDB).

### Analysis of drug sensitivity

We conducted an in-depth analysis of candidate diagnostic genes for renal fibrosis and drug sensitivity of anti-tumor drugs in an effort to find better new drugs for the treatment of renal fibrosis and develop more drugs to improve the condition. To be specific, gene expression data and drug sensitivity data were downloaded from the CellMiner database (https://discover.nci.nih.gov/cellminer/home.do) and 15 drugs FDA-approved or clinical trial drug were selected for study. The sensitivity analysis of potential prognostic genes to antitumor drugs was carried out using the R packages “impute,” “limma,” “ggplot2,” and “ggpubr” by Pearson correlation analysis.

### Cell culture and treatment

HK-2 cells obtained from the Chinese Academy of Sciences Cell Bank (Shanghai, China) were grown in DMEM/F12 media supplemented with 100 μg/mL. streptomycin, 100 U/mL penicillin, and 10% fetal bovine serum. Subsequently, HK-2 cells were given 20ng/ml TGF-β1 treatment for 48 hours to construct a fibrosis model *in vitro*.

### Reverse transcription-quantitative polymerase chain reaction (RT-qPCR)

In short, first of all, total RNA was extracted using the RNAiso Plus reagent from HK-2 cells that had been subjected to TGF-β1 or not, in accordance with the kit’s instructions (TaKaRa, Dalian, China). Secondly, using the HIScript RT SuperMix kit (Vazyme, Nanjing, China), cDNA was obtained. Afterwards, SYBR Green reagent (Vazyme, Nanjing, China) was used to conduct the RT-qPCR experiment following the kit’s instructions. Every sample was analyzed utilizing the 2-ΔΔCT value method. The specific primers were synthesized by Sangon (Shanghai, China). The reference used in this experiment was β-actin.

## Results

### Identification and enrichment analysis of EMT-related DEGs

**Figure 1:**
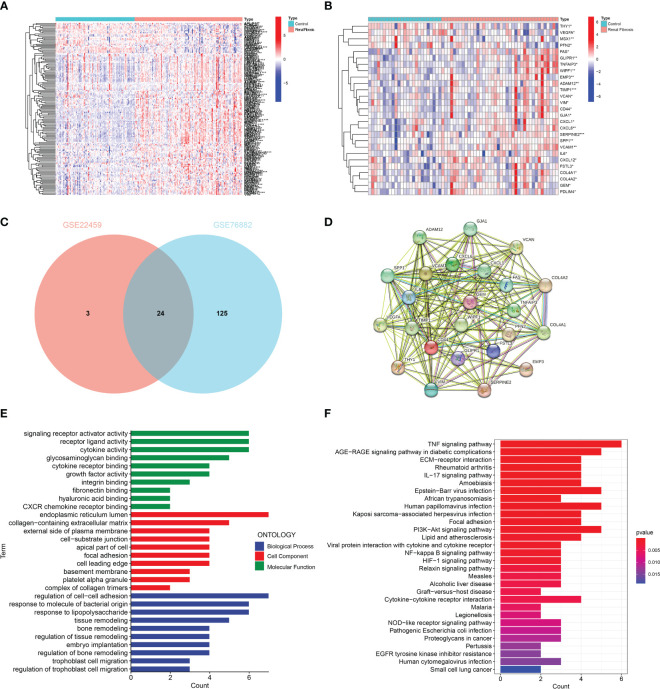
Identification and enrichment analysis of EMT-related DEGs. **(A)** Heat map of the ETM-related DEGs in GSE76882. **(B)** Heat map of EMT-related DEGs in GSE22459. **(C)** The Venn diagram showing the intersection of DEGs in GSE76882 and GSE22459. **(D)** PPI network of DEGs. **(E)** GO enrichment and **(F)** KEGG pathway enrichment analysis of DEGs.

Firstly, we screened 149 EMT-related genes in GSE76882 (**Figure 1A**) and 27 EMT-related genes in GSE22459 (**Figure 1B**) that were differentially expressed between renal fibrosis and control group by using the R package “limma”. Then, the intersection was picked to obtain 24 EMT-related DEGs (**Figure 1C**). A PPI network of these DEGs were constructed using the online website namely STRING (**Figure 1D**). In addition, an enrichment analysis of these DEGs was performed to explore their possible biological functions. GO enrichment analysis showed that these DEGs were mainly enriched in signal receptor activation activity, receptor ligand activity, cytokine activation, endoplasmic reticulum lumen, collagen-containing extracellular matrix, external side of plasma membrane, regulation of cell-cell adhesion, response to molecules of bacterial origin, and response to lipopolysaccharide in molecular functions, cell component, as well as biological processes (**Figure 1E**). KEGG enrichment analysis showed that these DEGs are mainly enriched in inflammatory, immune and oxidative stress-related pathways, such as TNF signaling pathway, AGE-RAGE signaling pathway in diabetic complications, ECM- Receptor interaction, and rheumatoid arthritics (**Figure 1F**).

### Identification of diagnostic markers for renal fibrosis

**Figure 2:**
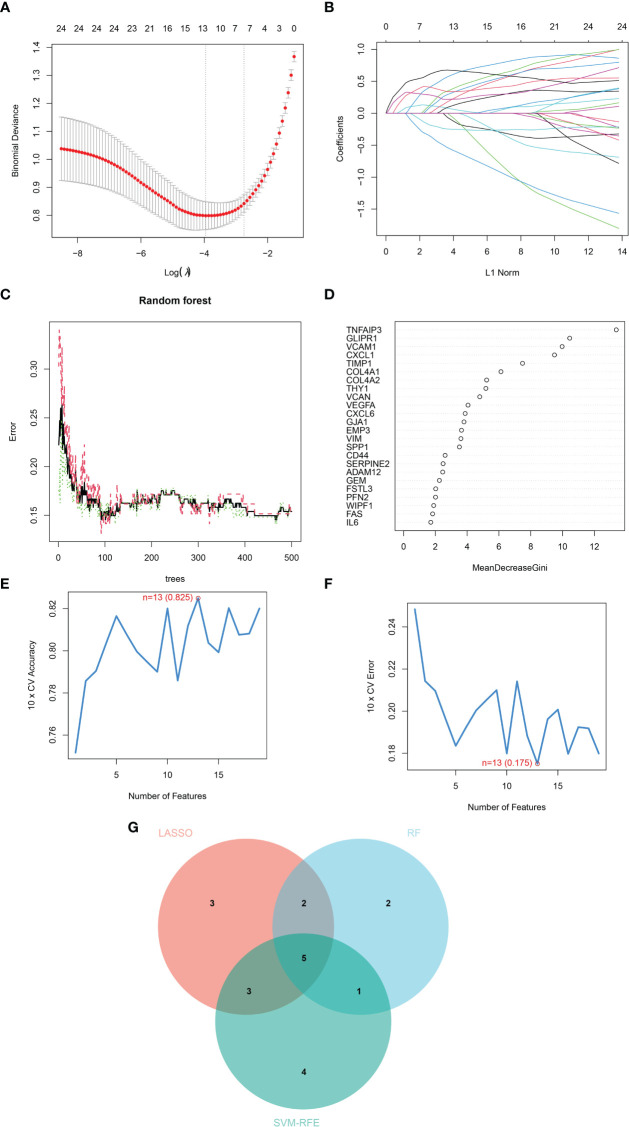
Identification of diagnostic markers for renal fibrosis by machine learning. **(A, B)** LASSO logistic regression, **(C, D)** RF **(E, F)** SVM-RFE algorithm screening diagnostic biomarkers for renal fibrosis. **(G)** The Venn diagram exhibiting the intersection of three machine learning models.

Three machine learning algorithms were manipulated to identify diagnostic markers for renal fibrosis. First, 13 possible biomarkers were revealed using the LASSO regression algorithm (**Figures 2A, B**). The top 10 obviously significant genes were analyzed by RF algorithm (**Figures 2C, D**). SVM-RFE analysis of EMT-related DEGs revealed a total of 13 genes in the model that could be used for diagnosis (**Figures 2E, F**). After that, we crossed the biomarkers obtained by the three machine learning algorithms, and obtained five common biomarkers, including COL4A2, CXCL1, TIMP1, VCAM1, and VEGFA (**Figure 2G**).

### Diagnostic power of five candidate biomarkers for renal fibrosis

**Figure 3:**
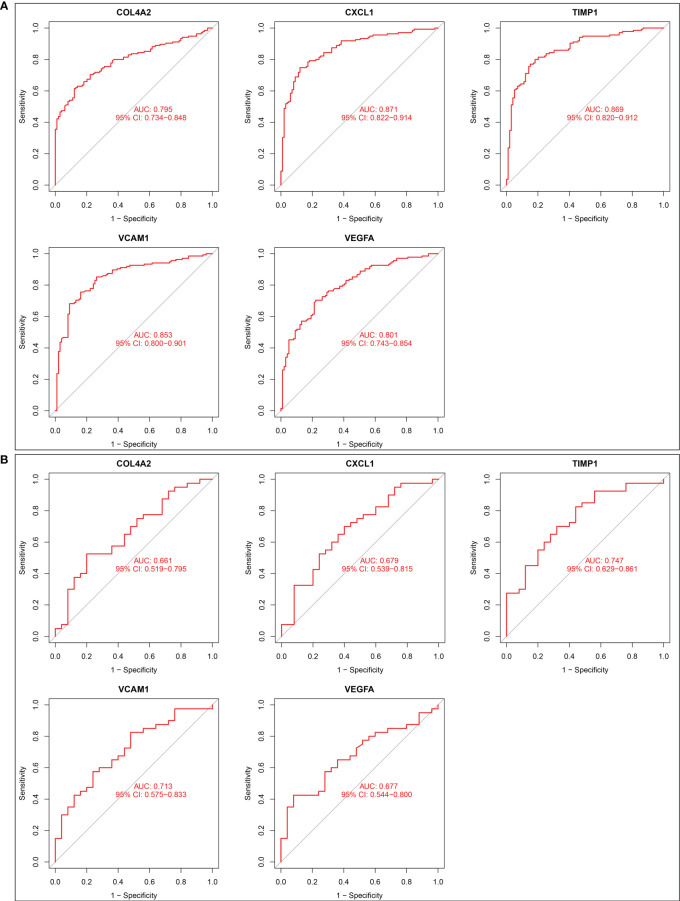
Diagnostic effect of five candidate biomarkers on renal fibrosis. **(A)** ROC curves of COL4A2, CXCL1, TIMP1, VCAM1, VEGFA in training set. **(B)** ROC curves of COL4A2, CXCL1, TIMP1, VCAM1, VEGFA in validation set.

ROC curves of five candidate biomarkers were drawn, and it was found that COL4A2, CXCL1, TIMP1, VCAM1, and VEGFA exhibited good diagnostic efficacy in both training dataset and validation dataset. The AUC values in the training set were 0.795, 0.871, 0.869, 0.853, and 0.801, respectively (**Figure 3A**). Diagnostic AUC values for COL4A2, CXCL1, TIMP1, VCAM1, and VEGFA were 0.661, 0.679, 0.747, 0.713, and 0.677, respectively, in the validation dataset (**Figure 3B**). These results indicate that these five genes have good diagnostic efficacy for renal fibrosis.

### Expression of five candidate biomarkers in renal fibrosis

**Figure 4:**
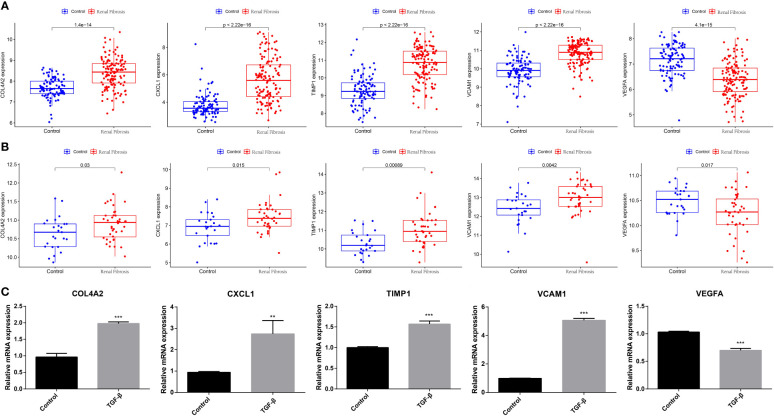
Expression analysis of five candidate biomarkers in renal fibrosis. **(A)** Expression levels of COL4A2, CXCL1, TIMP1, VCAM1 and VEGFA in the training set. **(B)** Expression levels of COL4A2, CXCL1, TIMP1, VCAM1 and VEGFA in the verification set. **(C)** RT-qPCR confirming the expression levels of COL4A2, CXCL1, TIMP1, VCAM1, and VEGFA in TGF -β1-treated HK-2 cells. “**” represented P <0.01, and “***” represented P <0.001.

**Figure 5:**
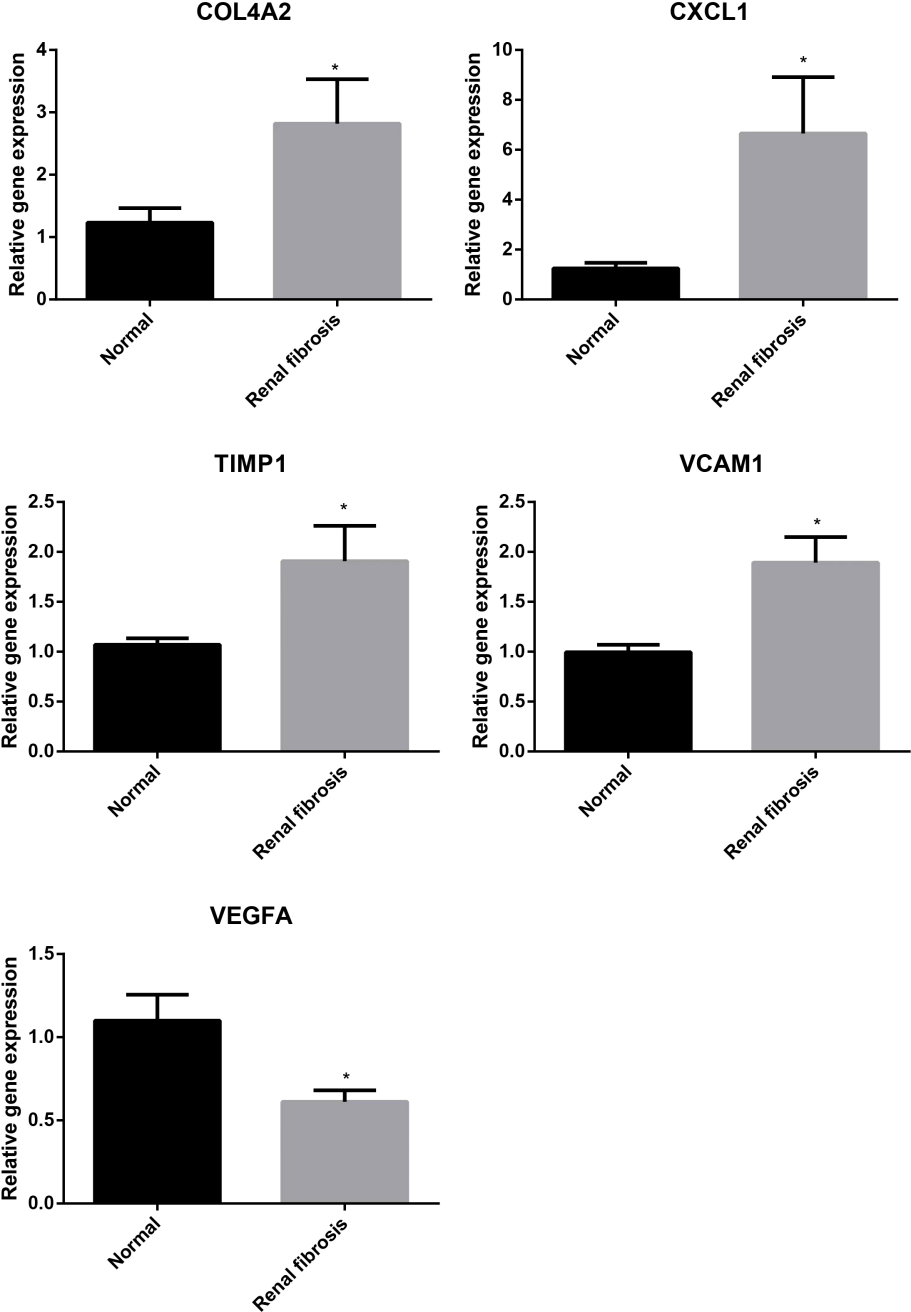
Validation of expression of diagnostic markers in blood samples from normal subjects and patients with renal fibrosis, with the results normalized by GAPDH. “*” represented P <0.05.

Next, we examined the expression of five potential diagnostic genes in the training set and validation dataset. As can be seen in **Figure 4A**, the expression of COL4A2, CXCL1, TIMP1, and VCAM1 was higher in the renal fibrosis group than in the control group, while the expression of VEGFA was lower. In the validation dataset, similar expressions were observed (**Figure 4B**). Additionally, RT-qPCR was employed for experimental confirmation, and it was discovered that the expression of COL4A2, CXCL1, TIMP1, and VCAM1 in HK-2 cells treated with TGF-β1 were also increased, while the expression of VEGFA was decreased (**Figure 4C**). In addition, the same was true for the mRNA expression of these genes in the blood samples as shown in (**Figure 5**). These findings were identical to the results of our bioinformatics analysis.

### Correlation between five candidate biomarkers and immune cells

**Figure 6:**
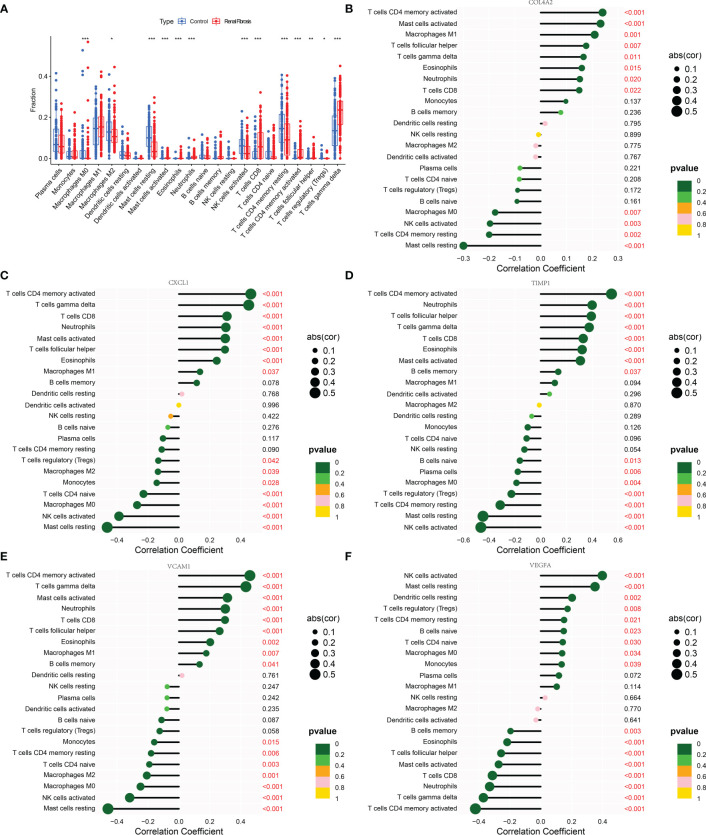
Correlation analysis between 5 candidate biomarkers and immune cells. **(A)** The box diagram showing the differences in various immune cells between the renal fibrosis group and the control group. **(B–F)** Bar graphs describing the correlation between COL4A2, CXCL1, TIMP1, VCAM1 and VEGFA and 22 kinds of immune cells. “*” represented P <0.05, “**” represented P <0.01, and “***” represented P <0.001.

The occurrence of renal fibrosis is often accompanied by a series of changes in the proportion and function of immune cells. In view of the role of immune cells in renal fibrosis, the relationship between five potential diagnostic genes and 22 different types of immune cells was studied. We discovered the renal fibrosis group had higher levels of mast cells activated, neutrophils, T cells CD8, T cells CD4 memory cells activated, T cells follicular helper, and T cells gamma delta, while macrophages M2, mast cells resting, NK cells activated, and CD4 memory resting content were lower than control group (**Figure 6A**). Further analysis demonstrated that COL4A2, CXCL1, TIMP1, and VCAM1 were positively correlated with T cells CD4 memory cells activated, mast cells activated, T cells follicular helper, T cells gamma delta, eosinophils, neutrophils and T cells CD8, and negatively associated with mast cells resting, NK cells activated and macrophages M0 (**Figures 6B–E**). In addition, COL4A2 was positively correlated with macrophage M1 and negatively correlated with T cells CD4 memory resting (**Figure 6B**). CXCL1 was also positively correlated with macrophages M1 and negatively correlated with monocytes, T cells CD4 naive, macrophages M2, and T cells regulatory (Treg) (**Figure 6C**). TIMP1 was also negatively correlated with Treg, plasma cells and B cells activated (**Figure 6D**). VCAM1 was also positively correlated with B cells memory, T cells CD4 memory resting, Treg, plasma cells, B cells naive, T cells CD4 naive, and monocytes negatively correlated (**Figure 6E**). Conversely, VEGFA is positively correlated with NK cells activated, mast cells resting, dendritic cells resting, Treg, T cells CD4 memory resting, B cells naive, T cells CD4 naive, macrophages M0 and monocytes, and negatively correlated with CD4 memory cells activated, T cells gamma delta, neutrophils, T cells CD8, mast cells activated, T cells follicular helper, eosinophils and B cells memory (**Figure 6F**).

### GSEA for five candidate diagnostic genes

**Figure 7:**
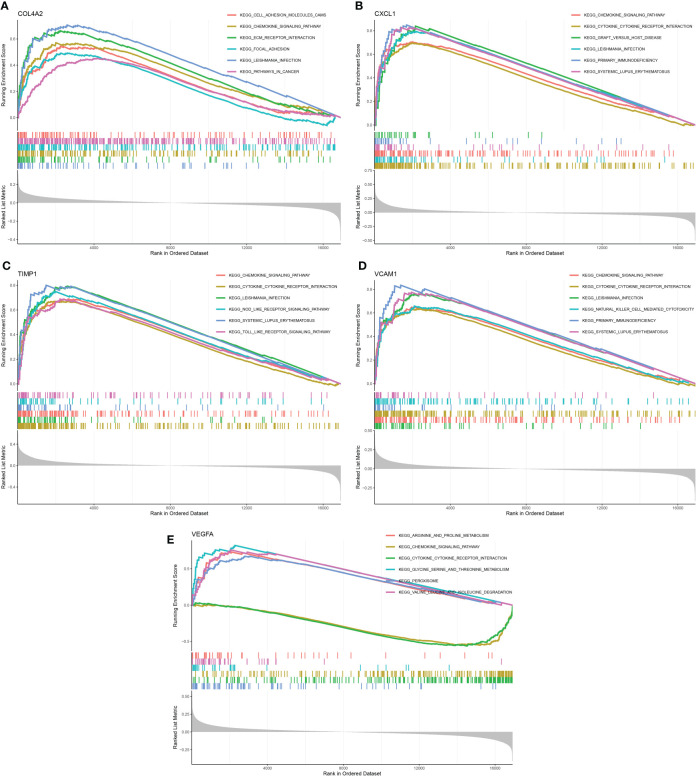
GSEA analysis of 5 candidate diagnostic genes. **(A)** The top six enrichment pathways of COL4A2. **(B)** The top six enrichment pathways of CXCL1. **(C)** The top six enrichment pathways of TIMP1. **(D)** The top six enrichment pathways of VCAM1. **(E)** The top six enrichment pathways of VEGFA.

GSEA was performed to investigate the potential biological roles of the five candidate diagnostic genes. The results indicated that COL4A2 was mainly involved in cell adhesion molecules, chemokine signaling pathway, ECM- receptor interaction, local adhesion, leishmania infection, and pathways in cancer (**Figure 7A**). Likewise, CXCL1 was mainly involved in chemokine signaling pathway, cytokine-cytokine receptor interaction, graft versus host disease, leishmania infection, primary immunodeficiency, and systemic lupus erythematosus (**Figure 7B**). TIMP1 was primarily involved in chemokine signaling pathway, cytokine-cytokine receptor interaction, leishmania infection, NOD-like-receptor signaling pathway, systemic lupus erythematosus, TOLL-like-receptor signaling pathway (**Figure 7C**). VCAM1 was predominantly involved in chemokine signaling pathway, cytokine-cytokine receptor interaction, leishmania infection, natural killer cell-mediated cytotoxicity, primary immunodeficiency, systemic lupus erythematosus (**Figure 7D**). VEGFA is principally involved in aging and proline metabolism, chemokine signaling pathway, cytokine-cytokine receptor interaction, glycine, serine, and threonine metabolism, peroxisome valine, leucine, and isoleucine metabolism (**Figure 7E**).

### Drug sensitivity analysis

**Figure 8:**
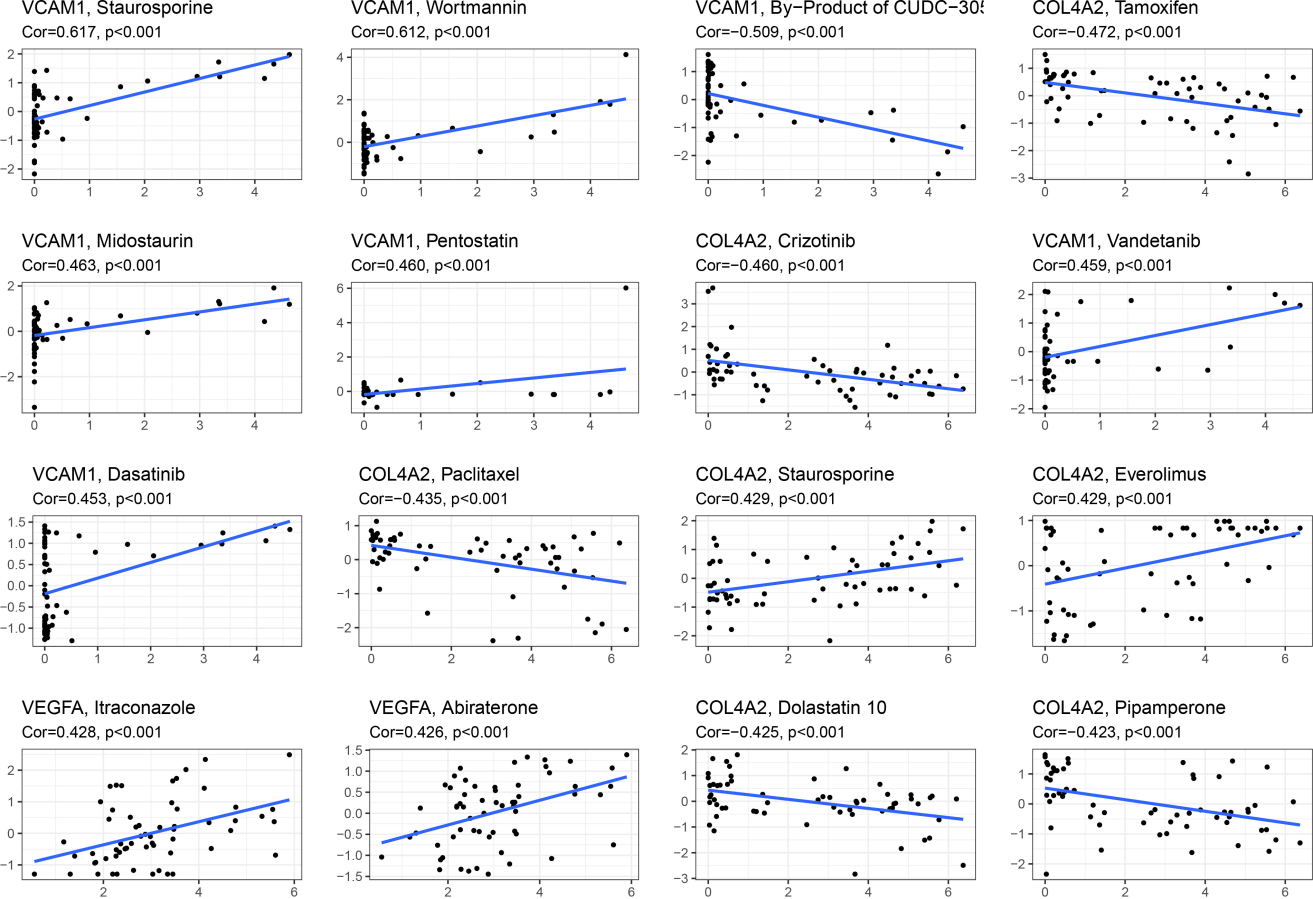
Relationship analysis between 5 candidate diagnostic genes and drug sensitivity. Gene and drug sensitivity are positively associated if Cor is more than 0; they are negatively correlated if Cor is less than 0.

In an effort to find better innovative drugs for the treatment of renal fibrosis and develop additional drugs to ameliorate the situation, in-depth analysis was conducted on candidate diagnostic genes for renal fibrosis and drug sensitivity of anti-tumor drugs. As shown in **Figure 8**, VCAM1 was positively correlated with staurosporine, wortmannin, midostaurin, pentostatin, and vandetanib, and negatively correlated with the CUDC-305. COL4A2 was positively correlated with staurosporine and everolimus, and negatively correlated with tamoxifen, crizotinib, paclitaxel, dolastatin, and pipamperone. VEGFA was positively correlated with itraconazole and abiraterone.

### Establishment of a nomogram for renal fibrosis

**Figure 9:**
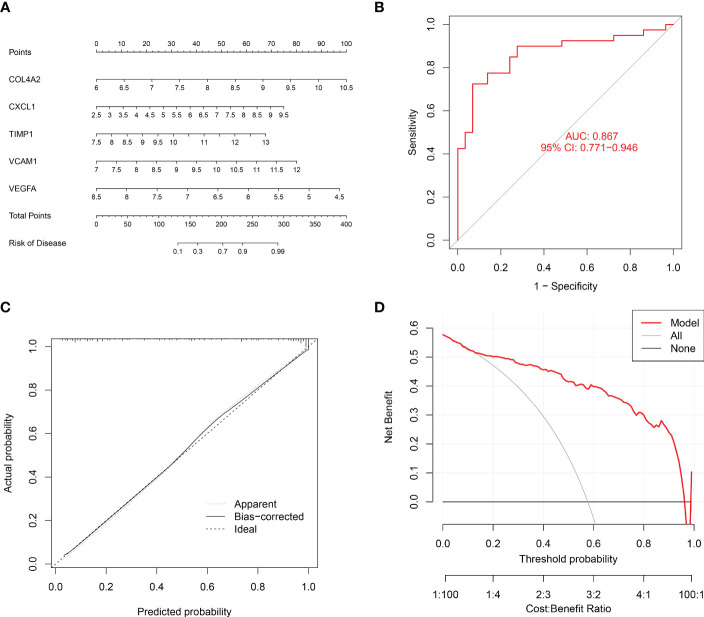
Establishment of a nomogram to diagnose renal fibrosis. **(A)** Nomogram for the diagnosis of renal fibrosis. **(B)** ROC curve for the combined diagnosis of five genes. **(C)** Calibration curve. **(D)** Decision curve analysis.

Finally, a nomogram was established to assess the risk of renal fibrosis (**Figure 9A**). A nomogram can transform independent prognostic risk factors into visual graphs for individualized prognostic evaluation. In this graph, each indicator axis indicates where the patient stands on each predictor variable scale using the five factors mentioned above. Each scale position has a corresponding prognostic point (top axis). The total score for each patient (bottom axis) is calculated, and the probability of disease is inferred from the bottom line. The ROC curve showed that the AUC value of this model was 0.867 (**Figure 9B**), indicating that this model had a very strong diagnostic effect. The 45-degree reference line and the actual line are the two main lines that make up the calibration curve. The apparent accuracy that hasn’t been calibrated for fit is represented by the dashed line, while the solid line is a nomogram performance of bootstrap correction and a scattering estimate for future accuracy. The calibration curves confirmed the efficacy of this nomogram model in predicting renal fibrosis (**Figure 9C**). Furthermore, a decision curve was generated to reflect the clinical value of nomogram models. In the decision curve, the y-axis and the x-axis represent the threshold probability and the net benefit, respectively. Decision analysis shows that this nomogram has a higher net benefit across a wide range of threshold probabilities, demonstrating the excellent predictability of the nomogram (**Figure 9D**).

## Discussion

As a significant characteristic of chronic kidney disease (CKD), renal fibrosis can lead to the loss of the damage of kidney structure and function (18). An increasing health burden is posed by CKD and renal fibrosis, which affect 10% of the world’s population (19). So far, numerous types of researches have shed light on the cellular and molecular mechanisms of renal fibrosis, but the strategies for diagnosing and treating this condition have not improved. Heat maps of DEGs were generated in this work using bioinformatics analysis of the GSE76882 and GSE22459 data sets. It was discovered that the former included 129 EMT-related DEGs, whereas the latter contained only 27 EMT-related DEGs. Intriguingly, the two data sets revealed a total of 24 common EMT-related DEGs. The intricate relationships of the different genes are clearly visible in the protein interaction network. Further research into the pertinent roles of DEGs and the signaling pathways that might be impacted revealed that DEGs might be crucial for signaling transduction, intercellular adhesion, and tissue remodeling. KEGG enrichment analysis elucidated that DEGs are involved in inflammatory response, proliferation, and immune-related pathways. Next, three machine learning algorithms described above were used to evaluate the DEGs to screen out five candidate biomarkers, including four significantly up-regulated genes, COL4A2, CXCL1, TIMP1, VCAM1, and one down-regulated gene, VEGFA, which were consistent with the results of *in vitro* RT-qPCR experiments.

Part of these genes have been linked to fibrosis, according to reports. Collagen type IV alpha 2 chain, which was encoded by COL4A2, is a key element of the basal membrane. Retinal hemorrhage is more common in people who have the COL4A2 mutation (20). There is a ton of evidence that certain organ fibrosis lesions are caused by aberrant type IV collagen deposition (21). Multitranscriptome study indicated that COL4A2 is a gene specifically associated with liver fibrosis and that it positively correlates with the development of hepatic fibrosis (22). In addition, prior research has demonstrated that COL4A2 is a possible biomarker for the diagnosis of acute liver failure (23). After infection or injury, mast cells and macrophages synthesize and release the neutrophil chemokine CXCL1/CXCL2 (CXC chemokine ligand 1/2) to trigger the early stage of neutrophil recruitment in response to inflammation (24). There is growing evidence that CXCL1 affects fibrosis in multiple organs. Bleomycin increased the release of CXCL1 in the lung fibrosis model (25, 26). The activation of NF- KB and TGF-Smad2/3 signaling by CXCL1 has been shown to mediate leukocyte recruitment, inflammatory response, and cardiomyocyte hypertrophy, promoting cardiac remodeling and fibrosis processes (27). Up-expression of CXCL1 exacerbates fibrosis mediated kidney damage, and inhibition of CXCL1 - CXCR2 shaft can greatly relieve kidney inflammation (28). Suppressing TIMP1 can alleviate hepatic fibrosis and myocardial fibrosis (29, 30). In diabetic rats, the levels of the fibrosis-related factors TGF-1, PDGF, and TIMP-1 can be decreased by renin-angiotensin-aldosterone system inhibitors, which can also be used to treat renal fibrosis (31). Increased TIMP1 in rats with aging-mediated renal interstitial fibrosis may be caused by antagonistic total saponins from Panax japonicas (32). The cell adhesion molecule VCAM1, which is present on the surface of endothelial cells and is secreted into the bloodstream, triggers a more extensive inflammatory response. According to research, individuals with systemic lupus erythematosus nephritis had urine levels of VCAM1 that were noticeably greater than those of healthy controls, indicating that VCAM1 may one day serve as a diagnostic tool for the condition (33). COL4A2, CXCL1, TIMP1, and VCAM1 have all been shown to be elevated in renal fibrosis, which is consistent with the findings of our experimental study. VCAM1 expression was markedly elevated in a fibrotic model of unilateral ureteral obstruction (34). By activating the HIF-1/VEGFA/VEGF receptor 1 (VEGFR1) signaling pathway and inducing the expression of the endogenous antioxidant superoxide dismutase 2 (SOD2) after unilateral ischemia-reperfusion injury, the antianemic drug FG4592 significantly reduced renal fibrosis and improved renal angiogenesis (35). Decreased pro-angiogenic factor vascular endothelial growth factor A (VEGFA) can cause glomerular microangiopathic and lead to the onset of pre-eclampsia, whereas upregulated VEGFA plays a protective role in diabetic nephropathy and polycystic nephropathy (36). Our research clarified the downregulation of VEGFA expression in renal fibrosis. Notably, one study found that patients with IgA nephropathy with high urinary VEGFA levels had a poor prognosis for renal replacement therapy (37). On the other hand, some studies have shown that increased VEGFA in certain development of fibrosis condition [32,33]. It is interesting that VEGFA has two distinctive expressions in fibrosis, and we will explore its mechanism in depth in future studies. However, the role of these genes in renal fibrosis remains unclear, which is the direction of future research.

Previous research has demonstrated that a variety of immune cells, including macrophages, T cells, and white blood cells, are involved in the regulation of renal fibrosis, and that depleting macrophages of eosinophils will result in a reduction in fibrosis (6, 38–40). CIBERSORT analysis revealed the types of immune cell infiltration, suggesting that the level and type of immune cell activation are key factors in renal fibrosis. In addition, further investigation revealed that COL4A2, CXCL1, TIMP1, and VCAM1 were all correlated with mast cells resting, NK cells active, and macrophages M0, as well as with T cells CD4 memory cells activated, T cells follicular helper, T cells gamma delta, eosinophils, neutrophils, and T cells CD8, the reverse is true with VEGFA. These results confirmed that COL4A2, CXCL1, TIMP1, and VCAM1 were positively correlated with some higher expressed immune cells in renal fibrosis, and VEGFA was positively correlated with some lower expressed immune cells in renal fibrosis. This suggested that these five genes may have an impact on renal fibrosis by regulating the activity of immune cells. Previous research discovered that deletion COL4A2 damages the transfer between T cells, preventing viral release, while COL4A mutations can prevent some immune cells from infiltrating (41, 42). Besides, CXCL1 was found to enhance macrophage invasion and migration, and its increased expression promoted neutrophil infiltration (43, 44). TIMP1 augmented macrophage migration and its transformation to M2 type (45). Reversely, IL10 induces dendritic cells to produce TIMP1 (46). Furthermore, anti-inflammatory M2-type macrophages down-regulate the expression of VCAM1 in endothelial cells (47). Knockdown of VEGFA blocked LPS-mediated M1-type macrophage polarization (48). These findings suggest that these five genes crosstalk with immune cells. Five putative diagnostic genes were mainly abundant in chemokine and cytokine-cytokine receptor signaling pathways, according to KEGG analysis.

Since renal fibrosis and cancer have a common feature, namely enhanced epithelial-mesenchymal transformation (EMT) (12), and many antitumor drugs have inhibitory effects on EMT (13–17, 49), we analyzed the relationship between these drug sensitivity and model genes to provide possible direction for the treatment of renal fibrosis. The results showed that VCAM1 was positively correlated with staurosporine, wortmannin, midostaurin, pentostatin, and vandetanib, negatively correlated with by-product of CUDC-305. COL4A2 was positively correlated with staurosporine and everolimus and was negatively correlated with tamoxifen, crizotinib, paclitaxel, dolastatin, pipamperone. VEGFA was positively correlated with itraconazole and abiraterone. These findings laid a foundation for anti-fibrosis drugs targeting COL4A2, VCAM1, and VEGFA. Furthermore, the AUC value demonstrated that each gene had an effective diagnostic effect. Finally, a nomogram was drawn to predict the risk of renal fibrosis. When using 5 genes for joint diagnosis, the AUC value was 0.867, indicating a good diagnostic value. The accuracy and reliability of this model in predicting the risk of renal fibrosis were verified by the calibration curve and the decision curve.

## Conclusion

In short, COL4A2, CXCL1, TIMP1, VCAM1, and VEGFA are promising diagnostic biomarkers of tissue and serum for renal fibrosis, which is helpful for the early diagnosis and treatment of patients with renal fibrosis.

## Data availability statement

The original contributions presented in the study are included in the article/**Supplementary Material**. Further inquiries can be directed to the corresponding authors.

## Author contributions

YGuo designed the study and performed most of the data analysis. ZY, ZH, and YGao performed data collection and curation. HG and HZ drawn pictures. KH, KC, and YM carried out PCR experiments. YB and designed and directed the study. XY designed the study, wrote and revised the manuscript. All authors contributed to the article and approved the submitted version.
